# Applying intervention mapping approach to a program for early intervention in first-episode mental crisis of a psychotic type

**DOI:** 10.1186/s41155-020-00141-0

**Published:** 2020-03-13

**Authors:** Daniela Martins Machado, Sheila Giardini Murta, Ileno Izídio da Costa

**Affiliations:** 1 Department of Clinical Psychology, University of Brasília, Campus Darcy Ribeiro, Brasília, DF 70910-900 Brazil; 2 Health Sciences School, Foundation for Teaching and Research in Health Sciences of the State Health Department of the Federal District, Brasilia, Brazil

**Keywords:** Intervention mapping protocol, Mental health, Non-ordinary states of consciousness, Intervention in crisis

## Abstract

The holotropic mind perspective, an integral part of the framework of transpersonal psychology, has been considered a revolutionary approach to a certain spectrum of experiences in Non-ordinary states of consciousness (NOSC) which conventional approaches tend to treat indiscriminately as pathological processes, because PHM recognizes in these experiences their healing and evolutionary potential. This article describes the needs assessment, implementation, and evaluation of an experiential and educational program on the holotropic mind perspective and its praxis, Holotropic Breathwork® (HB), with students and professionals from the Group for Early Intervention in First-Episode Mental Crisis of a Psychotic Type of the University of Brasilia. The intervention aimed to establish change goals and objectives that would promote the adoption of the holotropic mind perspective’s elements, such as a framework to broaden and strengthen mental health programs that assist people experiencing NOSC. The stages developed, inspired by the Intervention Mapping protocol, included a needs assessment; elaboration of change objective matrices; selection and description of methods based on theory and their applications; conception, planning, and implementation of the intervention; and results evaluation. Participants reported that the intervention allowed the expansion of their theoretical-conceptual and technical frameworks, giving them a less pathologizing understanding of and approach to NOSC and allowing them to perceive and manage such states, not as indiscriminately pathological expressions, but as phenomena inherent to the human condition that can be accepted and cared for without the exclusionary and exhaustive bias of mental disorders. Limitations and practical implications are discussed.

## Background

Human history presents, in every society and culture, a wide array of therapeutic approaches toward health and illness, including the psychological. The first investigations of psychology and psychiatry strongly emphasized the field of neuroscience, presuming that one could find in the body the causes of various psychological phenomena as well as the solutions to these problems—something that has only been reinforced by the advent of psychopharmacology (Costa, 2017; Shorter, 2008). While recognizing the importance of these early approaches, a large part of the theoretical and technical framework applied to the study and management of psychological phenomena is underpinned by the logic of the discourse between the normal and the pathological in mental health (Lopes, 2009). One may infer that the practice of pathologization of various psychological phenomena, observed and attested in the scientific literature (Wetzel, Pavani, Olschowsky, & Camatta, 2017), has its deepest roots in the absence, still, of epistemological and clinical instruments in the fields of traditional psychology and psychiatry that could offer non-pathologizing explanations or management. Their current theoretical-technical framework has exposed several psychological phenomena related specifically to experiences of non-ordinary states of consciousness (NOSC), explained today exclusively through the lens of illness.

In this scenario, the holotropic mind perspective (HMP), proposed by Czech psychiatrist Stanislav Grof, becomes important, as it advocates stepping back from this pathologization bias and taking a certain spectrum of NOSC experiences as qualitative changes that may hold developmental potential when well-managed and should not, a priori, be considered anomalous mental digressions (Grof, 1987, 2015). The researcher distinguishes, nonetheless, between the NOSC experiences that cause suffering and have no evolutionary potential and those which, with or without suffering, have the potential to be heuristic, transformational, and healing (Grof, 2015). Throughout 60 years of work and studies, the author, accompanied by a worldwide community of therapists and researchers, has been endorsing the dissemination of his theoretical framework and the outcomes of applying the technique developed by him, i.e., holotropic breathwork as a less pathologizing approach for the understanding, management, and therapeutic integration of NOSC experiences (Afanasenko, Emelianenko, & Emilianenko, 2014; Brewerton, Eyerman, Cappetta, & Mithoefer, 2012). As the value of a non-pathologizing approach to NOSC-connected psychological phenomena has been evidenced, the experience of the Group for Early Intervention in First-Episode Mental Crisis of a Psychotic Type (GIPSI) from the Center for Psychology Assistance and Study (CAEP) at the University of Brasilia (UnB) is highlighted. Established in 2001, this group is inspired by the British model of early intervention in psychosis to welcome and care for people experiencing NOSC, even when only prodromes are present, to minimize or interrupt the psychological aggravation that commonly occurs and which grows the longer first intervention is delayed (Allard, Lancaster, Clayton, Amos, & Birchwood, 2018; Marshall & Rathbone, 2011; Reichert & Jacobs, 2018; Schmidt et al., 2015).

Taking HMP as a starting point, the stages of an educational and experiential program around HMP and its praxis, HB, were recorded. HB’s development was inspired by the intervention mapping protocol and stresses the stages of needs assessment; the intervention’s expected results; selection and description of methods based on theory and their applications supported by evidence for their success; conception and implementation of the intervention carried out; and results evaluation (Bartholomew, Parcel, Kok, Gottlieb, & Fernández, 2011; Murta & Santos, 2015; Schaafsma, Stoffelen, Kok, & Curfs, 2013). Intervention mapping (IM) is a systematic approach to plan interventions used worldwide (De Lepeleere, Verloigne, Brown, Cardon, & De Bourdeaudhuij, 2018; Lamort-Bouché et al., 2018), which holds that health interventions based on theory and their applications tend to be more successful (Bartholomew et al., 2011). The intervention aimed to establish change goals and objectives that would promote the adoption of HMP elements, such as a framework to broaden and strengthen mental health programs that assist people having NOSC experiences.

This study has the purpose to describe the application of IM to the planning, implementation, and evaluation of an experiential and educational program on the holotropic mind perspective and its praxis, Holotropic Breathwork® (HB), with students and professionals from the Group for Early Intervention in First-Episode Mental Crisis of a Psychotic Type of the University of Brasilia.

## Method

**Fig. 1:**
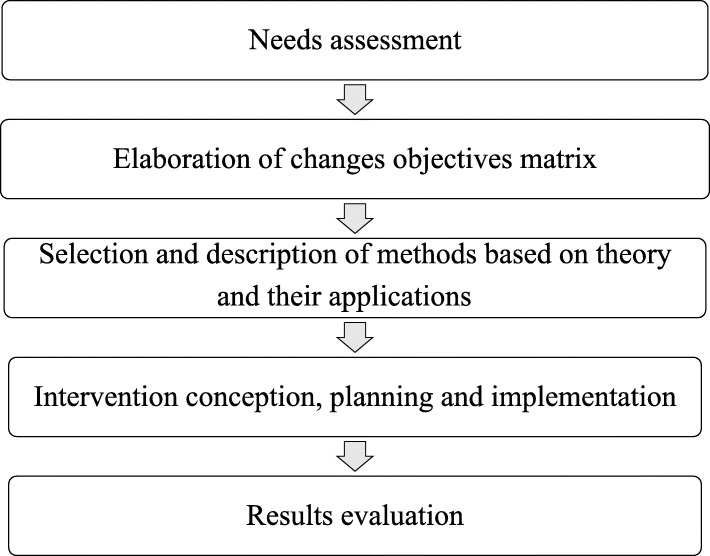
Flowchart of intervention planning, implementation and evaluation phases

The stages of the educational and experiential program in HMP that were performed inspired by IM, from needs assessment to results achieved, are presented as follows (Fig. 1). The study was approved by the Committee for Ethics in Human and Social Sciences of the University of Brasília.

### Needs assessment

In the needs assessment stage, the problem the intervention focuses on should be scrutinized, seeking an approximation with reality into which is inserted, along with the actors involved in it, the interactional dynamics that occur in the scenario, and related behaviors. This investigation can be carried out with qualitative methods, such as interviews or focus groups (Köche, 2016). This investigation’s results can be used in later stages to foster a better understanding of the intervention’s objective, the behavioral and environmental determinants involved in it, and greater clarity regarding the objectives of change and performance as well as to indicate the best intervention strategies and their possible outcomes (Bartholomew et al., 2011).

The sample who participated from needs assessment was composed by 30 people linked to GIPSI Program, among interns, volunteer professionals, and researchers. GIPSI is a Standing Extension Program of the Department of Clinical Psychology of the Institute of Psychology at the University of Brasilia. It consists of an interdisciplinary team of professionals, volunteers, interns, and researchers from the fields of psychology, psychiatry, nursing, social service, occupational therapy, and others. This program focuses on the welcoming and care of people in their first mental health crisis and their relatives as well as on the development of research related to severe psychological distress and its management (Freitas & Costa, 2018).

The needs assessment was performed via a set of methods (Kok, Peters, & Ruiter, 2017), including documental analysis, literature review, participant observations, and semi-structured interviews. Initially, a documental analysis consisting of the reading of the GIPSI Orientation Manual (GIPSI, 2018) and the results of internet searches for pages with information about the program, which returned the GIPSI Facebook Page (GIPSI, 2019) and UnB’s main website (http://www.unb.br).

The literature review, focused on the description of the GIPSI experience, consisted of searching using the string “*Grupo de Intervenção Precoce nas Primeiras Crises do Tipo Psicótica*.” The results from literature databases Brazilian Virtual Health Library-Psychology (BVS-Psicologia) and Brazilian Coordination Portal for the Improvement of Higher Education Personnel (Portal CAPES) was one article and, at Google Scholar, one article and one citation, whose original article was recovered at the Pluralities in Mental Health Journal website. For the observation and interview, a script was prepared, adapted from IM (Bartholomew et al., 2011; Rohrbach, 2014), which guided the observation of aspects of GIPSI Program management and the interview with a key informant (KI) in this program (Table 1). Participant observation took place at the welcoming meeting for new GIPSI team members. The program coordinators held a dialogued exhibition presenting the program history, mission, and overview as well as an evaluation of the previous year and perspectives for the current year, 2018. There was a presentation and then testimonials of group members, including professionals, volunteers, interns, and researchers. Observations were recorded in a logbook (Severino, 2017). The interview was conducted later by one of the professionals who works as a volunteer in the program, a KI by virtue of clinical experience, and time engaged at GIPSI. The strategy used for data collection was to send the script via the WhatsApp messaging application and receive the responses in audio files.

**Table 1:** Needs assessment in the GIPSI program

Observation script and interview	
1. How long have you been in the program?	
2. How would you describe GIPSI organizational structure?	
3. Which are the reasons why GIPSI was created?	
4. How do you describe, according to this program, psychological distress?	
5. What changes in the assistance for people in mental health crisis in the context of GIPSI?	
6. What would you change for the people treated within GIPSI?	
7. What does the GIPSI treatment consists of?	
8. What is the description of aspects in the early intervention model adopted by GIPSI?	
9. Who is part of the planning actions in the program?	
10. Are community members, patients, or family members involved in this planning?	
11. What are the theoretical approaches in psychology used by the service team?	
12. What are the possible difficulties and challenges to develop the program?	
13. How would you describe the strategies to evaluate the program?	

### Intervention expected results: changes objectives matrix

In this stage, based on the information acquired in the previous stage, the goal was to elaborate a matrix of interactions between performances objectives, change objectives, and behavioral determinants (Bartholomew et al., 2011; Murta & Santos, 2015; Schaafsma et al., 2013). The GIPSI members were considered the intervention’s priority target population and, as implementers, the first author and two HB facilitators, qualified and certified by the *Grof Transpersonal Training*® (GTT), were requested for this activity. The choice of these individuals was due to the nature of the work, developed by the GIPSI Program, in which they welcomed and followed-up with people in psychological distress, a phenomenon which, in the scope of this program, is taken as an integral aspect of existence. To be avoided at all costs is its a priori framing as a nosographic classification of mental disease. Instead, it seeks to understand it from a broader phenomenological perspective that suggests a more integrative management of these experiences (Costa, 2010). That, ultimately, is the clinical standing sought to be reinforced in the target group, based on HMP, especially as regards NOSC experiences. Here, the expected outcomes or desired changes were also listed for each change objective indicating determinants and related performance goals. This allowed, at a later stage, defining the best intervention strategies and their possible outcomes (Bartholomew et al., 2011; Kok, 2014). The goal of this intervention was reiterated as well through the inclusion by members of the GIPSI Program of HMP elements (Grof, 2015) in their clinical practice as a less pathologizing psychological approach to people who had experienced NOSC.

The psychological theories that analyze behavioral changes make the inseparability between those changes and behavioral determinants explicit, and this identification is indispensable to the effort undertaken to change a behavior or to adopt new behaviors. Determinants can be defined as “psychological variables or regulatory processes that are assumed, on the basis of empirical or theoretical evidence, to be causal antecedents of behavior” (Kok et al., 2016, p. 3). They can be of an emotional or cognitive nature and/or related to beliefs or, moreover, have their origin in automatic associations, be it at an individual level or with environmental resonance. The change objectives comprise precise changes in behaviors that are proposed based on their determinants. In this sense, this study identified cognitive determinants, of both an awareness order and a knowledge order, as well as attitudinal determinants of skills and self-efficacy. Each performance objective listed below is related to one of these determinants, through specific change objectives within which the professionals: (1) Assess their level of knowledge regarding different psychological approaches, including HMP; (2) demonstrate being open to a theoretical and experiential approach to HMP and its praxis, Holotropic Breathwork®; (3) indicate reasons for a theoretical and experiential approximation to HMP and its praxis; (4) decide to join the educational and experiential program on HMP and HB and evaluate their resonances with their health and quality of life; (5) participate in the project’s educational program, attending the workshops offered; (6) participate in the project’s experiential program, attending the HB workshops; and (7) include HMP aspects in their theoretical framework and in their clinical approach to people in mental health crisis. A description of the interactions between performance objectives, change objectives, and behavioral determinants is shown in Table 2. The parameters defined were the identification of participants according to Stanislav Grof’s explanatory model for NOSC phenomena and the adoption of elements from this perspective in the clinical approach of therapists. Such parameters were also the essential goal of this initiative.

**Table 2:** Matrix of interactions between performance objectives, change objectives, and behavioral determinants

Change Objectives by Determinants					
Performance Objectives	Awareness	Knowledge	Attitude	Self-efficacy	Skills
Therapists evaluate their level of knowledge regarding different psychological approaches, including Stanislav Grof's HMP (2015)	They recognize their tendencies towards traditional and contemporary approaches to psychology	They examine their knowledge of traditional and contemporary approaches to psychology			
They demonstrate being open to a theoretical and experiential approach to HMP and its praxis, Holotropic Breathwork (HB)	They become aware about the importance of broadening their personal theoretical-technical framework of psychological approaches	They express their prior knowledge of HMP and their HB practice			
They indicate reasons for a theoretical and experiential approach to HMP and its praxis, HB	They recognize the value of dialogue between different therapeutic approaches	They check knowledge gaps regarding HMP	They express intention and willingness to join the project's educational and experiential program	They express confidence in the length of the project's educational and experiential program stages	
They decide to participate in the educational and experiential program in HMP and HB and evaluate their resonances in their health and quality of life	They recognize the limited diversity of psychological approaches presented during their academic education	They list the main theories addressed during their professional background	They express commitment to the project's educational and experiential program		They organize their personal calendars to participate in project activities
They participate in the project's educational program, attending study meetings	They recognize the therapeutic value of the Grofian approach to mind in juxtaposing HMP with traditional approaches in psychology	They list the main topics of HMP, pointing out their expansions in relation to traditional approaches	They state they are optimistic about incorporating HMP knowledge into their clinical practice	They express confidence in participating in discussions about HMP and its praxis	They make individual investments in bibliographic and documentary research about HMP and its praxis
They participate in the project's experiential program, attending HB workshops	They recognize HB as an integrative and heuristic therapeutic strategy as they experience the HB process	They describe HB's praxis, justifying its procedures in relation to the theoretical framework that underpins it	They state they are optimistic about the therapeutic potential of HB praxis	They express confidence in the HB process	They actively participate in group discussions
					They actively participate in HB experiences
They include aspects of HMP in their theoretical framework and approach to people in mental crisis, experiencing NOSC	They recognize the therapeutic value of the Grofian approach to mind by incorporating its elements into clinical practice	They describe the elements of HMP that allow a less pathologizing approach to people experiencing NOSC	They express positive attitude towards incorporating HMP elements and their praxis into their clinical practice		They incorporate elements of HMP into the reception and management of cases in their clinical practice

Intervention goal: Inclusion by therapists (trainees, professionals and researchers) from the Group for Early Intervention in First Episode Psychosis-GIPSI of elements of the Holotropic Mind Perspective (HMP) as a less pathological psychological approach

### Selection and description of methods based on theory and their applications

In this stage, methods based on theory and evidence that can be used to change the behavior determinants, which have been evidenced and translated into performance objectives in previous stages, are selected. Here, all the conditions for the application of the selected methods must be ensured to promote efficacy. Next, the methods need to be translated into practical applications, which means effecting activities and materials, and adapting them to the specific target group and intervention context, in this case, GIPSI members. At this stage of the planning, the participation of target-group members and implementers is especially important (Peters, 2014). The methods proposed are the result of a literature review of their pertinence and evidence for efficiency when applied. In addition to scientific evidence, the researchers’ broad professional and academic experience in using the methods also contributed to their choice.

The related methods are mentioned as follows:

1. Active learning—a method that fosters learning through goal-oriented practice (Kelder, Hoelscher, & Perry, 2015; Kok et al., 2016) that has been applied through a mix of activities such as expository dialogue, which consists of an explanation focused on HMP during which theoretical support was offered for the articulation of knowledge, cognitive enrichment, debating of view, and experiences concerning the subject (Svinicki & Mckeachie, 2012); brainstorming—group activity that fosters the exploration and sharing of sensitive and rational material addressing the triggering theme (Svinicki & Mckeachie, 2012).
2. Cooperative learning—encompasses positive interdependence, face to face verbal interaction, individual responsibility, social skills, and task processing in group environments (Gillies, 2016; Johnson, Johnson, & Smith, 2014; Kok et al., 2016).
3. Self-evaluation—increases awareness of oneself and of one’s personal and affective cognitive performance in a practical and learning environment. It is based on the transtheoretical model of behavior change and encompasses the following stages: pre-contemplation, contemplation, preparation, action, and maintenance (Kok et al., 2016; Prochaska, Redding, & Evers, 2015).
4. Defining goals and gradual tasks—includes planning future activities related to the intended objectives and accepting tasks whose level of difficulty gradually increases. Both methods are based on the theory of self-regulation (Kelder et al., 2015; Kok et al., 2016).
5. Feedback—this method allows, based on learning objectives and reference criteria as well as self-analysis, evaluation and feedback between peers and the activity coordinator (Suskie, 2018). With this method, the offer of information was favored to participants in relation to their cognitive or practical performance, considering the activities proposed (Kelder et al., 2015; Kok et al., 2016).
6. Participation—this method assures high participant engagement in the discussion and decision-making for change actions (Cummings & Worley, 2015). It can be applied through roundtable discussions as a participative and problematizing strategy that fosters and promotes debate and collective knowledge construction (Machado & Moura, 2016).
7. Problematizing—this method abets the exercise of action-reflection-action, having extracted from reality and previous knowledge all means to acquire new knowledge and new skills. It has been widely applied in contexts of andragogy and popular education (Ausubel, 2008; Freire, 2011; Freire, 2014).
8. Literature review—a method of scientific research based on empirical studies, other literature review articles, theoretical articles, or experience reports, via database or abstract searches of periodicals that allows a theoretical deep-dive into a specific topic (Köche, 2016). It contributes to evidence-based practice (LoBiondo-Wood & Haber, 2017).
9. Holotropic Breathwork®—a method based on HMP (Grof, 2015). It is a mind-body practice developed in a therapeutic environment that involves a hyperventilation technique and is accompanied by evocative music to propitiate the attainment of heightened states of consciousness. Because of these states’ healing, transformative, and heuristic potential, they are called NOSC. The practice should be conducted by qualified professionals certified by *Grof Transpersonal Training®* (Afanasenko et al., 2014; Grof, 1987; Grof, 2015). A summary of the methods and their applications related to the determinants and change objectives to which they apply is set out in the Matrix of Change Objectives (Table 3).

**Table 3:** Matrix of interactions between determinants, change objectives, methods, and applications

Determinants and change objectives	Methods	Applications
Awareness of particular trends in traditional and contemporary approaches to psychology, as well as the importance and value of expanding their personal theoretical-technical framework on psychological approaches Knowledge of different traditional and contemporary approaches to psychology as well as the particular theoretical limitations regarding HMP and its praxis Attitude of intention and willingness to join the project's educational and experiential program Skill to present oneself as confident in fulfilling the educational and experiential program foreseen for the Project	• Participation • Problematization • Self-evaluation	Therapists evaluate their level of knowledge regarding different psychological approaches, including Stanislav Grof's HMP (Grof, 2015) They demonstrate being open to a theoretical and experiential approach to HMP and its praxis, HB They indicate reasons for a theoretical and experiential approach to HMP and its praxis, HB
Awareness of the limited diversity of psychological approaches presented in their academic background Knowledge of the main topics of HMP and their expansions relative to traditional approaches Attitude of commitment to the project's educational and experiential program Skill to organize their personal calendars to participate in project activities	• Active learning • Participation • Cooperative learning • Feedback • Setting goals and gradual tasks	They decide to participate in the educational and experiential program in HMP and HB and evaluate the resonances in their health and quality of life
Awareness of the therapeutic value of the Grofian approach to mind in confronting HMP with traditional approaches in psychology Knowledge of the main topics of HMP and their expansions in relation to traditional approaches Attitude of optimism about incorporating HMP knowledge into their clinical practice Skill of investing in bibliographic and documentary research about HMP and its praxis Skill to actively participate in group discussions	• Active learning • Literature review • Participation in conversation rounds • Cooperative learning • Feedback	They participate in the project's educational program, attending study meetings
Awareness of HB as an integrative and heuristic therapeutic strategy as they experience the HB process Knowledge of HB's praxis and its procedures in relation to the theoretical framework that underpins it Attitude of optimism about the therapeutic potential of HB praxis Skill to actively participate in HB experiences	• HB experience • Participation • Cooperative learning	They participate in the project's experiential program, attending HB workshops
Awareness of the therapeutic value of the Grofian approach to mind by incorporating its elements into their clinical practice Knowledge of the elements of HMP that allow a less pathologizing approach to people experiencing NOSC Positive attitude toward incorporating HMP elements and their praxis into their clinical practice Skill to incorporate elements of HMP into the reception and management of cases in their clinical practice	• Participation • Problematization • Self-evaluation • Cooperative learning • Feedback • Setting Goals and Gradual Tasks	They include aspects of HMP in their theoretical framework and approach to people in mental crisis, experiencing NOSC.

### Conception, planning, and implementation of the intervention

In this stage of the program development, the objective was to integrate practical applications within an organized plan in which all the elements already described in the previous steps are gathered (Bartholomew et al., 2011). Meetings were held with GIPSI members to introduce the proposal, making them aware of the theoretical and practical dimensions of the holotropic mind approach as well as of the educational and experiential program proposed. Later, this was also done with the Institute of Mental Health team. In this stage, strategies for the adoption, implementation, and sustainability of the program in the context of its application and target group were assessed (Kok et al., 2017). All resources necessary for the application of the methods, including facilities, furniture, audiovisual and sound resources, mats and cushions for the breathwork practice, stationery for the making of mandalas in the integration stage of the experiment, and other general-objective materials were secured and provided. Implementers and participants were mobilized with the aid of members of the intervention’s target group (Bartholomew et al., 2011; Kok, 2014; Stralen et al., 2008). A partnership between researchers linked to University of Brasilia and the Institute of Mental Health, a unit of the State Health Secretary of the Federal District, Brazil, was established. The terms of this partnership were the assignment of facilities to carry out the educational and experiential workshops provided for in the program in exchange for the inclusion of professionals from service careers in the activities to be developed in the program. All actors in this stage offered contributions to the improvement of the offerings and, later on, provided feedback about the development of the activities and their personal and environmental impacts. In the service, people who formed a support network for the work were identified, ensuring fluidity in the development of interventions.

### Results evaluation

The results evaluation stage included the development of an evaluation plan based on previous stages. The evaluation was directed at the process and results indicators (Bartholomew et al., 2011). As process indicators, early action adoption in the intervention plan was verified, highlighting the two meetings of the educational and experiential program during which expository dialogues focusing on the presentation of the holotropic mind perspective and HB practices were conducted. Result indicators were obtained through content analysis (Bardin, 2016) of what the participants’ said during the roundtable discussions, at the end of workshops, and their answers to the electronic interview sent by e-mail and WhatsApp to participants.

This interview included questions related to conceptions of psychological distress, NOSC experiences, theoretical anchorage of the participants’ clinical practice, and perceptions of the HMP presented during the workshops and its praxis, HB, which they could experience during the program implementation. The questions also encompassed the resonances in personal development and clinical practice of the new theoretical concepts around HMP and perceptions of the participants’ NOSC experiences experienced through the practice of HB. In this stage, returning to the established goal and to the performance and change objectives defined was indispensable for verifying their scope regarding their inclusion by the mental health program therapists of HMP elements in their clinical approach.

## Results and discussion

### Needs assessment

The analysis of the results evaluation data showed that GIPSI is a program consisting of a multidisciplinary team whose mission is to act early in welcoming and assisting people, and their families, in their first-episode mental crisis and to develop activities in consonance with the principles of universality, integrality, and equity of Brazil’s publicly funded health care system (GIPSI, 2018) and of the Brazilian Mental Health Law (Act No. 10,216, 2001). It is an expanded clinic that offers a distinct intervention that aims to provide the best support possible during the crisis (Freitas & Costa, 2018). GIPSI emerged around the problem of how to assist people undergoing severe psychological distress in situations of mental health crisis, understood here as psychosocial dislocation: a disruption or change in personal balance in effect up to this point requiring an intervention to assure the protection and well-being of the subjects until they return to states of equilibrium or less suffering (Costa, 2017; Schmidt et al., 2015).

Prior to its implementation, the program developers identified that, in the course of caring for people in mental health crisis, family and professionals often resort to hospital admittance, thus establishing a cycle of stigmatization, aggravation, and chronification of mental conditions that, if managed another way, would lead to less iatrogenic outcomes (Grof, 2015). In that sense, and based on the English model of early intervention in psychosis (Marshall & Rathbone, 2011; Reichert & Jacobs, 2018; Schmidt et al., 2015), a set of studies and early assistance for people with signs of psychological distress was implemented in 2001, taking as assumptions the observation that hospital admittance had not been shown to be the best option for those who present psychotic suffering prodromes and that proper and early management can interrupt the ongoing aggravating situation (Costa, 2017; Freitas & Costa, 2018). Clinical experience, including that of the University of Brasília’s Center for Psychological Assistance and Study, has revealed an open field of interventions for which no alternative was yet in place, as acute cases were often referred to mental health emergency services.

With that in mind, since its implementation, the program has been directed at people under severe psychological distress in their first-episode mental crisis of a psychotic type. Family participation has also been a criterion for inclusion in the program. The systemic theory (Costa, 2010) recommends broadening the scope of care from the individual to the family, as exemplified by the interviewee's perception that: “The suffering includes everyone involved, father, mother, child.” (KI). Criteria that excluded individuals from participating in the program were cases in which there are organic causes for the psychological situation (associated clinical aggravation) and cases related to psychoactive substance abuse. However, all cases are evaluated individually to determine whether they will be a part of the program or not.

Regarding the expected results of the program, as hospital admittance determinants were identified as an undesirable intervention (Barreto, 2014), efforts were made to avoid hospitalization as well as indiscriminate use of medication by offering individual and group therapy assistance in a personalized and humanized way. Weaknesses in the management of these clients were also identified, such as not including families in the therapeutic care. The program has been assisting the families in a concomitant and integrated way to care for patients. In this sense, management based on relevant and changing determinants and without hospitalization and in a humanized and integrated way with crisis reversal and family support is a priority outcome of the program. As for the investigation of methods based on theory and evidence and their application concerning the program, a variety of therapeutic strategies starting with a 24-h crisis hotline are used. The utilization of face-to-face reception, as opposed to screening, is based on the National Humanization Policy of the Brazilian Ministry of Health (Ministério da Saúde, 2006), which promotes the use of listening and understanding in order to learn the meanings assigned by the subject to his own experience while simultaneously allowing the participants a dialogical construction of new meanings (Lévy, 2001).

This strategy avoids a priori psychologizing or psychopathologizing the subject’s demands and experiences and invests instead in the phenomenological exercise of understanding and acting in the realm of experiences and relationships (Freitas & Costa, 2018). According to the perception of the KI, the diagnostic classification systems: “[...] do not allow comprehension of the phenomenon’s whole complexity” (KI). The use of systems such as the Diagnostic and Statistical Manual of Mental Disorders (DSM-5) or the International Statistical Classification of Diseases and Related Health Problems (ICD-10) circumscribes not only one’s reading of psychological phenomena but also of therapeutic intervention.

Moreover, in the course of psychotherapeutic care, approaches from different schools are used, such as psychoanalysis, gestalt, cognitive behavioral, and psychodrama, according to the therapist’s familiarity with these therapeutic techniques. Furthermore, philosophical approaches to care and professional ethics and complexity theory are also considered. In the program evaluation, it was clear that there was no involvement of members of the community or the target group (patients and relatives) in the initial planning actions of GIPSI. However, these groups have a privileged space to talk during the therapeutic process and group activities, in which they “can offer their impressions,” having been invited to evaluate the care and the propositions for improvement of the initiatives connected to the program.

Regarding the program development, it was observed that the model of early intervention in psychotic crises developed by GIPSI, albeit inspired by the English experience of early intervention in psychotic crisis (Marshall & Rathbone, 2011; Reichert & Jacobs, 2018; Schmidt et al., 2015), distances itself from this model while advancing some of its aspects, notably abandoning the “psychosis” diagnosis and evaluation based on prodromes of this pathology to look at the signs of disorganization and suffering that could indicate a mental health crisis (Freitas & Costa, 2018). Another peculiarity of the GIPSI Program is the inclusion of the family in the therapeutic care based on the understanding that suffering reverberates from the individual into their family environment. Experiments prior to the constitution of the program were carried out when the group was created. They were coordinated by the program’s creator and academics and graduate students participated as well. The aim was to conduct studies of severe psychological distress and crisis response. The consolidation of the program arose from this experience (Freitas & Costa, 2018; Oliveira, 2011).

As for the program’s feasibility, GIPSI’s offerings consist of a crisis hotline, an interprofessional welcoming of people in their first severe mental crisis, psychotherapy and individual and family guidance, psychiatric consultation, social and occupational assistance in the care unit or outside it, and psychosocial and diagnostic evaluation. During the program’s presentation, it was emphasized that the secrecy and confidentiality of customer identities and information are safeguarded in accordance with ethical standards (GIPSI, 2018).

Among the barriers to the program’s development, the precariousness of the psychosocial care network of the Federal District, which lacks sufficient psychosocial assistance centers thus resulting in GIPSI being overloaded, was cited. As barriers, paradigm divergences in the assistance model were noted, as the caregivers entering the program often bring from their background a hospital and medical perspective concerning the management of people in mental health crisis. It was mentioned that approximately 20% of the people and families who begin their treatment in the program quit, making adherence a challenge. Regarding these challenges, the interviewee reveals:

There are many challenges, one of the main is related to public policies for mental health services. [The Federal District] is still one of the worst in the national ranking. Another problem is that there are professionals who still have a view more related to hospitalization in all cases. (KI)

Some other challenges are posed for the program, such as those related to interdisciplinary and interprofessional work, integration between theory and practice, and the need for openness to a paradigmatic deconstruction. To overcome the identified barriers, the program proposes methodological workshops to discuss aspects of the program’s structure, process, concepts and results, and experiential workshops in which relational aspects between group members and care with the caregiver are addressed. These workshops are held every 6 months and can be organized on special dates if there is a need for technical and methodological alignment. In addition to this strategy, the whole team undergoes individual and collective supervision by the program staff. This includes the evaluation of the work process, the survey of limitations and needs, the sharing of experiences overcoming barriers, and the formulation of propositions to overcome them.

Regarding the program’s evaluation methods, it was observed that GIPSI members use internal and external strategies. In addition to the previously mentioned workshops and team supervision, evaluation has been carried out through assessment by research funding organizations, such as the Brazilian Coordination for the Improvement of Higher Education Personnel (CAPES). Also, through publications—be they books, articles, theses, or dissertations—GIPSI members raise visibility of the program experience and boost its recognition by the scientific and care-giving communities. One final evaluative strategy is the organization of scientific and cultural events, where this small community presents its products and services, open to public judgment. Today, around 40 people work at GIPSI, including professionals, volunteers, interns, researchers, and graduate students (masters and doctorate candidates). The evaluation of the GIPSI program, considering the steps proposed for the planning and development of health interventions, allowed greater appropriation of the context in which the educational and experiential program in HMP and its praxis, HB, to be developed.

### Intervention

Based on the needs survey and according to the planning established, the envisaged educational and experiential program was conducted. Telephone and e-mail communication were carried out with facilitators certified by Grof Transpersonal Training*®* to formalize the invitation to conduct HB workshops. Plane tickets and lodging were provided as the implementers lived outside Brasilia. All equipment and materials needed to fulfill the educational and experiential program were provided. The partnership with the Institute of Mental Health for the assignment of the facilities was secured after a visit to the unit, and formalization of the reservation request was made via the Federal District Health Authority’s Electronic Information System. As a counterpart to the partnership with the institute, vacancies were reserved for service professionals and volunteers interested in the holotropic approach. Visits to GIPSI and the institute were made during team meetings to present the educational and experiential program, including awareness-raising and invitations to participate in the initiative.

The educational and experiential program took place in two workshops, the first on April 26–28 and the second on June 15–17, 2018. The structure of the workshops was identical. On the first day of each workshop, the activities were focused on expository dialogues (Svinicki & Mckeachie, 2012), conducted by the first author, in two sessions of approximately 2 h each during which they presented HMP concepts and fundamentals and HB practice as well as the architecture of mental disorders according to HMP. Following the expository part of both workshops, roundtable discussions were conducted, lasting a maximum of 2 h, during which participants made suggestions, asked questions, and received guidance on the experiential activities in HB to be developed in the following days. The HB sessions took place throughout the second and third days of each workshop, followed by an integration activity through plastic expression (mandala making) and discussion circles about the experiences, for a total of two 10-h sessions of activities in each workshop. In the program’s activity schedule, 12 h were allocated for educational activities and 40 h for experiential activities, adding up to 56 h of activities in the two workshops, as detailed in Table 4. The first workshop was attended by 19 people: ten professionals, five students, and four volunteers. In the second, there were 20 people: ten professionals, three students, and seven volunteers. Seven professionals, one student, and three volunteers participated in both workshops.

**Table 4:** Schedule of program activities

Workshop period						
	1st Workshop—April 2018			2nd Workshop—June 2018		
	1st day	2nd day	3rd day	1st day	2nd day	3rd day
Time						
8–11 am		Breathwork	Breathwork		Breathwork	Breathwork
11 am–12 pm		Integration	Integration		Integration	Integration
2–5 pm	Expository Dialogue	Breathwork	Breathwork	Expository Dialogue	Breathwork	Breathwork
5–6 pm		Integration	Integration		Integration	Integration
6–8 pm	Roundtable	Roundtable	Roundtable	Roundtable	Roundtable	Roundtable
Duration	6 h	10 h	10 h	6 h	10 h	10 h

### Intervention outcomes evaluation

To begin the evaluation of intervention outcomes, it should be noted that of the 28 people who participated in the workshops, 22 of them answered the interviews, of which 11 were professionals, seven were students, and four were volunteers. For the objectives of the following analyses, it was decided not to use with the volunteers’ responses as they were not clinical practitioners and thus lack this point of view. In fact, of the 18 professional (P) and student (S) respondents, only five (27.75%) reported they had had experienced NOSC before the HB practice offered in the program. As for the NOSC experiences that occurred during the HB workshops, all 18 (100%) respondents stated that it was a transformational experience that impacted their personal and professional development:

After the experience with holotropic breathwork, I carried out two group therapy sessions and managed to conduct the session more serenely than usual, finding ways to work with what is common to the group as a whole in an easier way." (P3)

"It was for me an experience that promotes self-knowledge and development." (P4)

"I had a huge increase in my personal analysis from my holotropic breathwork experiences. From these experiences, I managed to advance my therapeutic process a lot. It is much more intense and is giving very nice results. (P11)

We recognize that the realization of the present educational and experiential program, built upon and inspired by the Intervention Mapping Protocol, was successful in choosing and implementing the methods and their outcomes. Active learning methods (Kelder et al., 2015; Kok et al., 2016; Machado & Moura, 2016), cooperative learning (Svinicki & Mckeachie, 2012), and participation (Cummings & Worley, 2015), applied through strategies such as expository dialogue, roundtable discussions, and the experience of the HB practice, allowed participants’ learning to be guided according to the established goals and with fruitful sharing of knowledge and therapeutic practices. These were common among and unique to the participants and included concepts and fundamentals regarding HMP and its praxis, HB, in an innovative way within the group: “It was a moment of great self-enlightenment. First, we had a theoretical exposition, shared our expectations and introduced each other and the concepts.” (P3)

The program provided an environment for participants to express themselves regarding the evaluation of the activities developed as well as for self-evaluation (Kok et al., 2016; Prochaska et al., 2015) concerning personal enjoyment rather than the content offered in that context. The evaluation process led to reflections on the design and assumed goals and gradual tasks (Kelder et al., 2015; Kok et al., 2016) with significant raising the awareness of everyone about the adoption of HMP elements in future clinical practice:

I feel much closer to people [...] This whole process has been important to me and I see great progress in my relationship with everyone. In my therapeutic work, in my relationships with clients, and with colleagues in supervision, I improved considerably in quality and personal confidence. (P11)

The discussion roundtables provided feedback (Suskie, 2018) and self-regulation time (Kelder et al., 2015; Kok et al., 2016), enriching all the participants as well as the group of implementers regarding how the program was conducted.

The practice of HB (Grof, 2015) has showed itself to be a method as well as a channel for problematization (Ausubel, 2008; Freire, 2011; Freire, 2014), mainly through experimentation by the groups of participants:

In addition to the therapeutic potential experienced, specifically triggered by HB, I think it is even more important to share that the experience as a whole, taking into account the people present, the facilitators, and the place, allowed me to experience a place of relaxation, lack of control, madness, ugliness, weirdness, outburst, beauty, ecstasy, and catharsis without any judgments. There was a feeling that I was allowed to express the way my organism felt it needed to be expressed that enabled me to take advantage of the therapeutic potential of the technique [...] The value that the experience provided as a whole was that I could be who I needed to be at that moment, with my body, with my bioenergy, and the more sincere, the greater the healing potential (P8)

The practice of HB has successfully allowed the exercise of action-reflection-action within the explanatory model of mind proposed by psychiatrist and researcher Stanislav Grof, wherein the existence of an expanded mental cartography includes remembrance levels beyond that of biographical memory, commonly studied by the traditional psychological schools. These different levels in the perinatal and transpersonal remembrance dimensions can be accessed when experiencing, in a spontaneous or induced way, NOSC. Such experiences, when well-received in an appropriate therapeutic environment, as proposed by the practice of HB, can be promoters of healing and personal development (Afanasenko et al., 2014; Grof, 1987; Grof, 2015; Rhinewine & Williams, 2007).

At this point in the analysis, resuming the action planning, it can be stated that the development of this educational and experiential program achieved the proposed goals, resulting in 16 (88.8%) of the respondents recognizing that the holotropic mind perspective is a less pathologizing approach to NOSC phenomena than conventional psychological approaches, which is represented in the following statements:

A powerful healing and self-encounter tool, capable of fostering inner peace, boosting self-esteem, and rescuing the spiritual dimension. (P3)

Yes, mainly for not judging the psychological phenomena that need to be manifested. (P8)

Undoubtedly, first, it does not treat psychological demands as diseases, but as part of the human being. In addition, it refers a lot to the phenomenological approach that brings us precisely this idea of going against the pathologization of mental suffering. (S1)

Only two (11.2%) of the respondents did not comment on this topic. Seventeen (94.35%) respondents agree with the statement that the practice of HB is a valid, important, and recommendable mental health tool for people who experience NOSCs. Here are some statements regarding HB practice:

Very interesting technique of self-knowledge, making it possible to experience deep levels of consciousness, as I observed. (P4)

The other conventional practices in mental health do not have such an ability to access our subconscious mind so assertively, to the point that we ourselves are the authors of our emotional and physical healings. (S1)

One (5.55%) of the respondents did not categorically state a standing:

I could not come to a 100% conclusion. (P1)

Of the 18 professionals and students respondents, 17 (94.35%) expressed interest in deepening their knowledge and willingness to include elements of HMP in their theoretical framework and clinical practice with people reporting NOSC experiences. The following statements are representative:

I will use many of its elements. It certainly prepared me better to welcome the user. (P5)

In fact, the perinatal stages (levels reminiscent of the Grofian perspective) are something to pay attention to; I have even reflected a lot on how this (re)birth process reverberates in my own life. (P8)

I would consider other dimensions, such as the perinatal and the perspective regarding the subject's relationship with reality. (P10)

In particular those [elements] that understand spiritual emergencies and the transpersonal scope of consciousness may be very relevant in dealing with the 'unspeakable' suffering of the people I treat in the future (S7)

Concluding the evaluation of the educational and experiential program implemented by us and underpinned by the IM protocol, it is notable that the participants’ acquisition of HMP allowed the expansion of their theoretical-conceptual and technical frameworks, making them aware of a less pathologizing understanding of and approach to NOSC phenomena. This in turn allowed the perception and management of such phenomena, not indiscriminately as pathological expressions, but as phenomena inherent to the human condition that can be accepted and cared for without the exclusionary and exhaustive bias of mental disorders.

### Limitations

One of the limitations of the educational and experiential program described here is the fact that the needs assessment for the GIPSI Program was conducted based on the few documents available in academic databases, one participant observation session, and one interview, though it was with a key informant. The low adherence of GIPSI Program members to the initiative, the target group of our study, also set a limitation. Although it was overcome by the inclusion of members of the ISM mental health program, and, in the end, the results of the initiative were broader and more positive than those initially envisioned, as two mental health programs were included in the process, rather than the single one originally planned.

## Conclusion

The experience of using the IM approach was of crucial importance for the success achieved by the initiative as it enabled the program to foster knowledge capable of guiding a clinical and social practice that offers greater support and care to the subjects and collectives in mental health care programs, such as those of GIPSI and ISM. The present exercise of using the Intervention Mapping protocol instrumentalized, in a less intuitive way, our intervention propositions, grounding them in theoretical and methodological bases and in evidence for their successful practical applications. Likewise, the unconstrained validation of participants of the value of the holotropic mind perspective shows that these contributions can be added to those already achieved by clinical psychology, expanding theoretical support for the deconstruction of discourse between normalcy and pathology, favoring a more respectful and dignified treatment of people who experience unique psychological phenomena such as NOSC.

## Data Availability

Data are available from the first author (email: daniluzmartins@gmail.com).

## Abbreviations

CAEP: Center for Psychology Assistance and Study
CAPES: Brazilian Coordination for the Improvement of Higher Education Personnel
GIPSI: Group for Early Intervention in First-Episode Mental Crisis of a Psychotic Type
GTT: Grof Transpersonal Training®
HB: Holotropic Breathwork®
HMP: Holotropic mind perspective
IM: Intervention mapping protocol
KI: Key informant
NOSC: Non-ordinary states of consciousness
UnB: University of Brasilia
